# The Probiotic Effects of *Lactobacillus brevis* CD2 on Caries Related Variables of Dental Plaque Biofilm

**DOI:** 10.1016/j.identj.2025.02.018

**Published:** 2025-03-26

**Authors:** Guglielmo Campus, Maria Grazia Cagetti, Anna Lehrkinder, Ali Alshabeeb, Nicole Caimoni, Peter Lingström

**Affiliations:** a Department of Cariology, Institute of Odontology, Sahlgrenska Academy, University of Gothenburg, Gothenburg, Sweden; b Department of Oral and Maxillofacial Sciences, Sapienza University of Rome, Rome, Italy; c Department of Biomedical, Surgical and Dental Sciences, University of Milan, Milan, Italy; d Asst Valle Olona, Dental Unit, Gallarate, Italy

**Keywords:** Probiotic, Sugar, Acidogenicity, In vitro, In vivo, Lactobacillus brevis CD2, Oral health

## Abstract

**Objectives:**

This study was based on the research question: “Does *L. brevis CD2* have an effect on the acidogenicity of sugar-exposed bacteria? To solve this question, a multistep study was planned: first, an in vitro investigation aimed to assess the acid production of monoculture bacterial solutions; and second, an ex vivo experiment to evaluate the production or inhibition of acids from plaque samples.

**Methods:**

*L.* brevis CD2 and several control strains (*Lactobacillus brevis CD2, Lactobacillus reuteri DSM 17938, Lactobacillus rhamnosus LB21, Lactobacillus plantarum 931, Streptococcus mutans Ingbritt*) were tested with various sugars; pH changes were recorded at specific time points using a micro-pH electrode. Additionally, for the ex vivo phase, the same sugars were added to equal amounts of pooled plaque from 9 healthy subjects with bacterial suspensions, as well as a control solution, and pH was monitored for up to 90 minutes. For the ex vivo phase, 9 adults were randomised in a crossover design for 28 days. For the in vivo phase, 26 healthy subjects used 1/2 lozenges 3 times daily containing either *L. brevis* CD2 (active) or no probiotic bacteria (placebo). Plaque acidogenicity was assessed using the microtouch method after a 10 ml mouth rinse containing 10% sucrose for 1 minute (on day 0 and day 28).

**Results:**

*L. brevis* CD2 exhibited the highest ability to inhibit the fermentation of fructose, lactose, and sucrose compared to the control strains (*P* < .05). A significant reduction in plaque acidogenicity was observed in vivo from day 0 to day 28 in the test group (*P* < .05).

**Conclusions:**

This study indicates that *L. brevis* CD2 mitgates the acidogenic attributes of plaque biofilm organisma in vitro*,* in vivo and ex vivo, suggesting its potential benefit as a caries preventive probiotic agent.

## Introduction

There is growing interest in the potential effects of probiotics on oral diseases and dental issues. A key property of probiotics^1^^,^^2^ is their ability to displace pathogenic microorganisms, potentially preventing or reducing oral disease and discomfort. By modulating the resident oral microbiota through the administration of probiotic products, probiotics may offer an additional strategy for the prevention and treatment of oral diseases. In the context of oral health, foods containing live microorganisms may alter the biofilm environment towards less acidic plaque.^3^ Various strains of *Lactobacillus* have demonstrated the ability to inhibit the growth of *Streptococcus mutans*, although the effect depends on factors such as the specific strain, colonisation sequence, and growth condition.^4^ For instance, the probiotic strain *Lactococcus lactis* produces bacteriocins with antimicrobial activity against periodontopathogens.^5^ Similarly, *Streptococcus A12* disrupts the bacteriocin production by *S. mutans* by producing challisin-like protease, while *Streptococcus dentisani* produces bacteriocins that target cariogenic bacteria and raise plaque pH *via* the arginolytic pathway, potentially hinding the formation of mature plaque.^6^ Dental caries, being a multi-factorial disease, is primarily driven by a microbial shift toward aciduric and acidogenic bacteria that metabolise fermentable carbohydrates.^7^, ^8^, ^9^ Several in vitro studies have explored the mechanisms by which probiotics may prevent caries, focusing on biomechanical properties such as coaggregation,^10^^,^^11^ the ability to bind to oral structures,^12^ and metabolic activity.^13^, ^14^, ^15^ The role of probiotics in the oral cavity can be summarised by their direct interactions with the oral biofilm—such as inhibiting pathogen adhesion and collagenase activity, competitive exclusion of bacterial binding sites, and participation in substrate metabolism. Probiotics also indirectly influence oral health by regulating mucosal permeability and preventing plaque formation through the neutralisation of free electrons.^13^^,^^14^

It is well known, that *mutans Streptococci* and caries-associated *Lactobacilli spp.* are significant microbial risk factors for caries.^16^ However, strategies aiming to eliminate cariogenic microorganisms, which are part of the endogenous microbiota, are not feasible and may be also imprudent.^17^ As a result, the administration of probiotics to replace at least part of the pathogenic microflora has been proposed as a potential preventive strategy.^10^

Several short-term clinical studies have evaluated the inhibitory effects of various probiotic strains on *Streptococcus mutans* and *Lactobacillus spp.*^18^, ^19^, ^20^, ^21^ These studies focused on the replacement of cariogenic bacteria with noncariogenic counterparts. Current literature, notwithstanding, provides limited data on the effect of probiotics on the acidogenicity of dental plaque following probiotic regimens.^19^, ^20^, ^21^, ^22^

*Lactobacillus brevis* CD2, whose systemic health benefits are also well established, is one of the probiotic strains suggested for caries prevention.^20^^,^^21^ The beneficial effects of *L. brevis CD2* on oral health has been reported in several studies.^23^^,-^^25^ This probiotic has been shown to reduce plaque acidogenicity and the salivary levels of *mutans Streptococci* in children at high risk of caries,^20^ and to improve caries-related risk factors and gingivitis in children with type 1 diabetes.^21^ Additionally, *L. brevis* CD2 has been shown to reduce the persistence of traumatic oral lesions in patients with fixed orthodontic appliances by almost 50%.^25^ However, when administered as lozenges, it did not prevent radiation-induced mucositis in patients undergoing treatment for head and neck cancer.^26^ To date, no studies have examined *L. brevis* CD2′s ability to produce or inhibit acid in human dental plaque.

Based on the existing literature, the research question addressed in this study is: “Is *L. brevis* CD2 able to have an effect on the acidogenicity of sugar-exposed bacteria? The null hypothesis is that *L. brevis CD2* is not able to modify plaque acidogenicity.

To solve the research question, a multistep study was planned: first, an in vitro investigation aimed to assess the acid production of monoculture bacterial solutions; second, an ex vivo experiment to evaluate the production or inhibition of acids from plaque samples. Two hypotheses were proposed: (1) there is no difference in acid production between *L. brevis* CD2, *L. reuteri* DSM 17938, *L. rhamnosus* LB21, *L. plantarum* 931, and *S. mutans* Ingbritt and (2) the probiotic strains do not inhibit the sugars/sugar alcohols fermentation by dental plaque. Third, the in vivo trial was planned to assess the effect of frequent oral administration of *Lactobacillus brevis CD2* on caries-related variables. The null hypothesis was that the use of *L. brevis* CD2 or a placebo would not result in any significant differences in plaque acidogenicity, plaque formation, or the salivary and plaque microbiota.

## Materials and methods

**Fig. 1:**
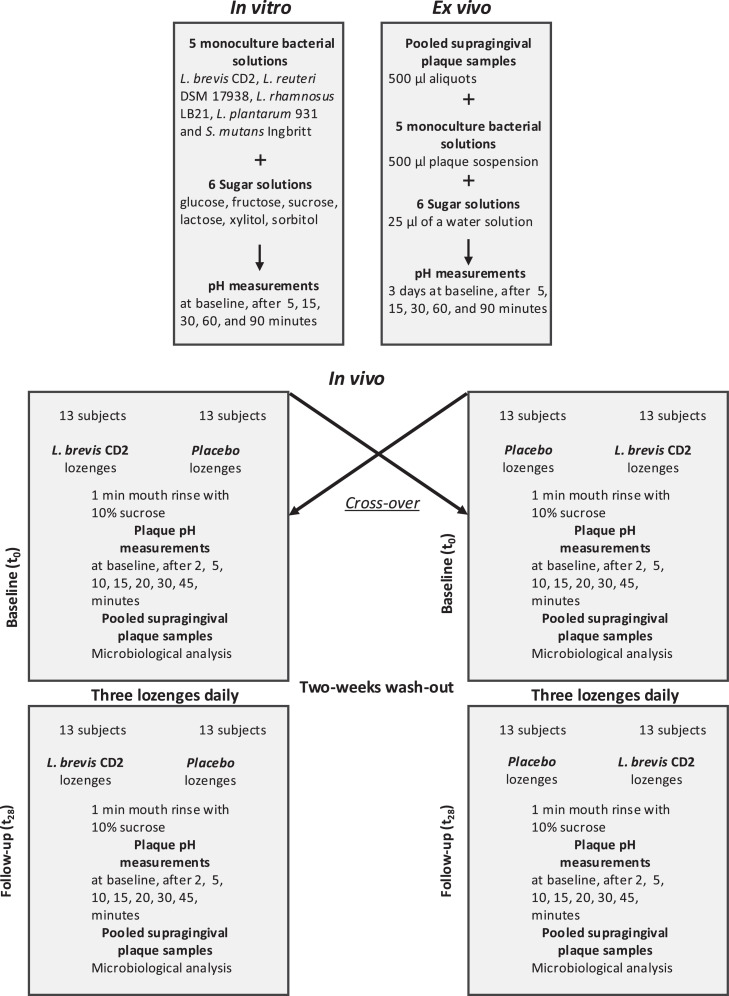
Flow-chart of the study.

The study was conducted at the Department of Cariology, Institute of Odontology at University of Gothenburg, Sweden. The study protocol was designed and drafted in accordance with the Helsinki Declaration of Human Rights and approved by the Gothenburg Regional Ethics Review Board (260-18). The design and the description of the study is displayed in Figure 1.

### In vitro phase

This phase was conducted in 2 parts using a double-blind, randomised design. Both the sugars/sugar alcohols and the bacterial strains were tested in a randomised order. The bacterial and sugar codes were sealed by an independent monitor and remained unopened until statistical analysis was complete.

### Bacterial strains and samples

The following bacterial strains were included: (1) *Lactobacillus brevis* CD2 (Inersan, Actial Farmaceutica/VLS), (2) *Lactobacillus reuteri* DSM 17938 (ProTectis, BioGaia, Stockholm, Sweden), (3) *Lactobacillus rhamnosus* LB21 (Essum AB), (4) *Lactobacillus plantarum* 931 (Essum AB), and (5) *Streptococcus mutans* Ingbritt (laboratory strain used as a positive control).

Each bacterial strain was cultivated on blood agar under anaerobic conditions for 24 hours at 37°C. The bacterial cells were then suspended and homogenised in sterile Fermentation Minimal Medium (FMM, pH = 7.4) and washed twice by centrifugation at 3500 rpm for 5 minutes, adjusting the optical density to OD = at 480 nm.

### Sugars and sugar alcohols

The following 4 dietary sugars were tested: (1) glucose (Merck, Damstadt, Germany), (2) fructose (Merck, Damstadt, Germany), (3) sucrose (Merck, Damstadt, Germany), and (4) lactose (Merck, Damstadt, Germany). Two sugar alcohols were also tested: (1) xylitol (Merck, Damstadt, Germany) and (2) sorbitol (Merck, Damstadt, Germany). An aliquot of 25 µL of a water solution of each sugar at the concentration of 500 nM was added to each bacterial suspension to initiate sugar fermentation.

### pH-measurements of bacterial suspension after sugar addition

pH measurements were conducted over 3 days, in 5 series performed daily. Each series included 6 Eppendorf tubes each containing 1000 µL of bacterial suspension. The tubes were incubated at 37°C for 1 hour. At baseline, the 6 different sugars were added in a randomised order for each series. pH measurements were taken at baseline and at 5 additional time points (5, 15, 30, 60, and 90 minutes) using a micro-pH meter (Orion ROSS Micro pH electrode Orion 8220BNWP).^26^

### Ex vivo phase

Nine healthy subjects (5 males and 4 females, aged 24-64 years) participated in the study; all volunteers signed informed consent prior to enrolment. Participants’ buffering capacity was 5.16 ± 1.30, salivary mutans *Streptococci* count was 5.28 ± 4.87 log10 CFU/mL, and lactobacilli count was 4.11 ± 4.25 log10 CFU/mL. Participants were instructed to refrain from brushing their teeth for 48 hours before the examination and to refrain from eating or drinking for 1 hour before the test. During 3 oral examinations 1 week apart, supragingival plaque samples were collected between 8 and 9 AM. using a sterilised dental carver and fluoride-free dental floss (REACH, waxed floss, Jonsson & Johnson, Canada).^27^ Pooled plaque samples were collected from buccal, lingual, and approximal sites of each subject. These samples were mixed and then suspended and homogenised in FMM by sonication for 5 seconds. The optical density was adjusted to 1 at 480 nm. The plaque solution was kept on ice for a maximum of 45 minutes before further processing. The 1000 µL aliquots were pre-incubated at 37°C for 1 hour. pH-measurements were carried out over 3 days, with 5 series performed daily. Each series consisted of 6 Eppendorf tubes: one with 1000 µL plaque as a control, and 5 with a 500 µL mixture of plaque suspension and 500 µL of each probiotic strain suspension. The tubes were incubated again at 37°C for 1 hour. At baseline, an aliquot of 25 µL of a water solution of each sugar was added in randomised order to the 5 series, except for the control, which received no sugar. pH measurements were taken at baseline and at 5 additional time points (5, 15, 30, 60, and 90 minutes) using the same micro-pH meter. All tests were run in duplicate.

## In vivo phase

### Sample size and participants

This phase followed a randomised, double-blind, placebo-controlled, cross-over design. Computerised randomisation was used for group assignment. Power analysis was conducted *a priori* for repeated measures ANOVA with an effect size of 0.3^20^ and a power of 0.95. This analysis suggested that 26 participants were required, with 13 subjects per group.

Twenty-six healthy adult subjects were included in the study, with the following inclusion criteria: (1) in good general health, (2) not regular users of probiotics, (3) not undergoing antibiotic therapy in the last 2 months, and (4) Capable of reducing plaque pH by at least 1 unit after a 10% sucrose mouth rinse.

All subjects came first for a screening visit to the clinic for a regular dental examination and plaque pH registration. The subjects will be identified among patients and staff at the university clinic as well as via announcement on the bulletin board. All volunteers signed an informed consent form before enrolment and participated in 2 periods, each lasting 4 weeks (28 days), in a randomised order (Figure 1). A washout period of 4 weeks preceded the first period, and there was another washout period between the 2 test periods.

Professional tooth cleaning was performed using a rubber cup and prophylactic paste (Cleaning RDA 170; CCS Clean Chemical) at the beginning of both washout periods, as well as after the data collection on day 0. Prior to each visit, subjects were instructed to refrain from approximal cleaning for the previous 48 hours, tooth brushing for the last 24 hours, and eating or drinking for the last hour before the visit. During the trial, participants were instructed to slowly suck on 3 lozenges daily—one after breakfast, one after lunch, and one in the evening before going to bed—while avoiding eating or drinking for 2 hours afterward. Throughout the trial, all participants were provided with a toothpaste containing 1450 ppm fluoride (Actavis AB) and a soft toothbrush. They were also advised not to use any other probiotic or sugar alcohol-containing products, and to avoid any other topical or professional fluoride treatments throughout the study.

### Test products

The following 2 products were used: (1) a lozenge containing *L. brevis* CD2 (active) and (2) a lozenge without *L. brevis* CD2 (placebo). The products were identical except for the lactobacilli content and were distributed in coded packages. The code was not broken until the data analyses was completed. The *L. brevis*-containing lozenges contained the following ingredients: lyophilised bacteria (200 mg), fructose (400 mg), mannitol (1020 mg), aspartame (40 mg), talc (60 mg), aerosol 200 (10 mg), PEG 6000 (50 mg), magnesium stearate (20 mg), tartaric acid (100 mg), and orange flavour (100 mg)*.*

### Acidogenicity of dental plaque

Plaque pH was measured at 2 interproximal sites (15-16 and 25-46) using an iridium microelectrode (Beetrode, MEPH-1; W.P. Instruments).^28^ pH-measurements were taken at point 0 (baseline), 2, 5, 10, 15, 20, 30, and 45 minutes following a 1-minute mouth rinse with 10% sucrose.

### Plaque index

The plaque index was assessed according to the Silness-Löe index.^29^ The facial/buccal, lingual/palatal, and approximal surfaces of 6 teeth (16, 12, 24, 36, 32, and 44) were stained with erythrosin (Rondell Red). Each surface was scored from 0 to 3, and the mean score of all surfaces was calculated.

### Microbial samples and analyses

Pooled plaque samples were collected by sterile toothpicks at 2 interproximal sites (14-15 and 24-45) and put into VMG II and processed for microbial analysis.^30^ The samples were dispersed by sonication for 10 s. After serial dilutions in 0.05 M phosphate buffer (pH7.3), 25 µL portions were plated on Mitis Salivarius Bacitracin (MSB) agar, Rogosa SL agar, and blood agar plates for *mutans Streptococci* (MS), *Lactobacilli* (LB) and total count (TC), respectively. Total streptococci (TS) were cultivated on MS agar plate under the same conditions as mutans *Streptococci*. After incubation, microorganisms were identified by colony morphology. The concentration of microorganisms was expressed as percentage of the total number of viable bacteria.

### Outcomes and dependent variables

The outcomes for the in vitro phase were bacterial sugar fermentation by means of pH measurements and the dependent variables were the type of bacteria, type of sugars, and the different time points. For the ex vivo phase the outcomes were the pH measurements, streptococci, and lactobacilli count of the pooled plaque depending on the type of sugars and probiotic strain suspensions. For the in vivo phase the outcomes were acidogenicity of dental plaque and plaque index and the dependent variables were examined evaluating the use of the lozenges with *L. brevis CD2* or the placebo and the corresponding effect on MSB, MS, and LB agar.

### Statistical analyses

Descriptive and analytical analyses of all data were performed. Descriptive statistics (means, standard deviations, 95% CI) were calculated for the 2 duplicate tests (or for glucose, triplicate tests). Data were analysed for statistically significant differences using repeated 1-way and 2-way analyses of variance (ANOVA). A 2-way factorial ANOVA was applied to assess differences in means across the various bacterial strains, sugars, sugar alcohols, and time points. The area under the pH curve at pH 5.7 (AUC_5.7_) for all the phases and pH 6.2 (AUC_6.2_) for the in vivo phase were calculated using Prism software (GraphPad Software). A *P*-value of <.05 was considered statistically significant.

## Results

### In vitro

**Fig. 2:**
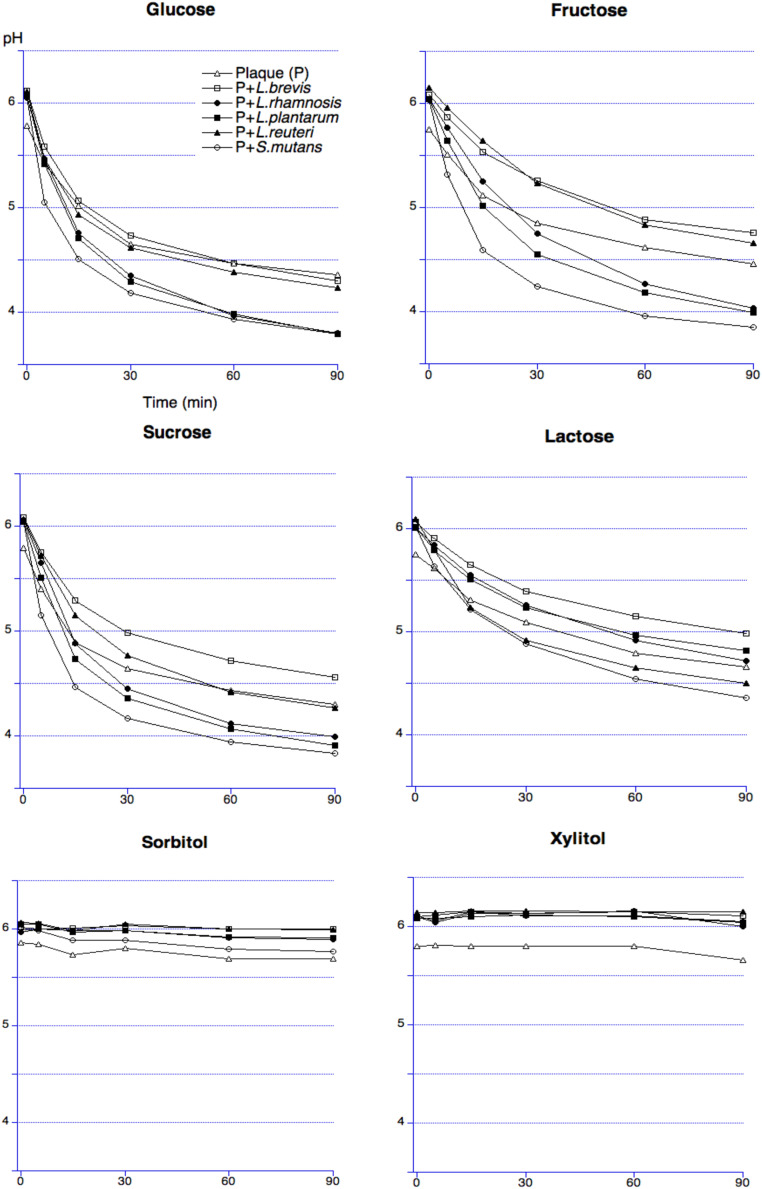
pH curves of different bacterial solutions and/or pooled dental plaque during 90 minutes after sugars exposure.

The baseline pH was between 6.7 and 6.8 for all bacterial suspensions, except for L. *reuteri,* which was approximately 7.0. The acid production ability varied depending on probiotic strain and sugar. For glucose, *L. brevis* and *L. reuteri* exhibited the least pronounced pH drop, while for sucrose, *L. reuteri, L. plantarum* and *L. rhamnosus* exhibited similar results. For fructose, the suspension of *L. reuteri* showed the smallest pH decrease, while for lactose, it was *L. plantarum* (Table 1). None of the bacteria were capable to ferment xylitol. A similar pattern was observed when analysing the area under the curve (AUC) of the pH in relation to the baseline value and the maximum pH drop (Table 1). The differences were statistically significant within each sugar group for the different bacterial strains (*P* < .01 for all strains). The minimum pH drop showed also statistically significant variations between the sugars, except for xylitol (*P* = .07). When ranking the AUC and maximal pH fall, *L. brevis* and *L. reuteri* exhibited the least pronounced pH decreases. Regarding the sugar fermentation, *L. brevis* CD2 showed the most pronounced pH fall with glucose, followed by fructose, sucrose, and lactose (Figure 2). Sugar alcohols produced minimal pH changes.

**Table 1:** Maximum pH fall and area under the curve (AUC_5.7_) expressed as mean ± SD of the bacterial suspensions after sugar addition with the rank order of different bacterial solutions for respective sugar solution after 90 minutes. Two-way factorial ANOVA was performed.

Maximum pH fall (mean ± SD) Two-way factorial ANOVA	*number of observation 42 R-squared = 0.67 F = 4.82 P < .01*					
Bacterial strain	Glucose	Fructose	Sucrose	Lactose	Sorbitol	Xylitol
*L. brevis* CD2	2.35 ± 0.90 (1)†	2.11 ± 1.20 (2)	1.79 ± 1.54 (4)	1.20 ± 0.91 (2)	0.47 ± 0.50 (2)	0.04 ± 0.07 (1)
*L. reuteri* SDM 17938	2.66 ± 0.07 (2)	0.64 ± 0.31 (1)	0.52 ± 0.17 (1)	1.70 ± 1.18 (3)	0.19 ± 0.07 (1)	0.12 ± 0.03 (5)
*L. plantarum 931*	3.15 ± 0.03 (4)	2.96 ± 0.01 (3)	0.65 ± 0.08 (2)	0.43 ± 0.12 (1)	0.85 ± 0.13 (4)	0.1 ± 0.02 (4)
*L. rhamnosus* LB21	3.14 ± 0.13 (3)	3.00 ± 0.16 (4)	0.90 ± 0.11 (3)	1.73 ± 0.49 (4)	0.72 ± 0.09 (3)	0.06 ± 0.05 (3)
*S. mutans* Ingbritt	3.31 ± 0.06 (5)	3.31 ± 0.03 (5)	3.32 ± 0.01 (5)	2.84 ± 0.01 (5)	1.49 ± 0.01 (5)	0.07 ± 0.04 (2)
*P-value*	<.05					.07

AUC_5.7_ (mean ± SD) Two-way factorial ANOVA	*number of observation 42 R-squared = 0.52 F = 3.00 P < .01*					
Bacterial strain	Glucose	Fructose	Sucrose	Lactose	Sorbitol	Xylitol

*L. brevis* CD2	163.60±94.63 (1)*	135.80 ± 91.50 (2)	124.70 ±54.04 (4)	75.79 ± 110.39 (3)	28.47 ± 27.75 (2)	3.20 ± 4.16 (1)
*L. reuteri* SDM 17938	181.70 ± 5.17 (2)	33.14 ± 1.97 (1)	31.15 ± 12.36 (1)	113.40 ± 79.80 (4)	16.49 ± 0.09 (1)	8.28 ± 1.07 (4)
*L. plantarum 931*	241.70 ± 2.97 (3)	209.40 ± 4.03 (4)	46.82 ± 3.30 (2)	26.87 ± 6.48 (1)	49.25 ± 11.74 (3)	6.33 ± 2.07 (3)
*L. rhamnosus* LB21	246.70 ± 11.46 (4)	203.80 ± 5.66 (3)	53.37 ± 3.83 (3)	89.25 ± 27.00 (2)	54.22 ± 2.14 (4)	3.99 ± 4.36 (2)
*S. mutans* Ingbritt	271.30 ± 3.96 (5)	259.90 ± 1.77 (5)	267.60 ± 1.20 (5)	204.20 ± 3.11 (5)	104.50 ± 2.40 (5)	20.47 ± 3.52 (5)
*P-value*	*<.05*					

⁎ Ranking order of AUC_5.7_ (mean ± SD) for respective sugar solution.

† Ranking order of maximal pH drop (mean ± SD) for respective sugar solution.

**Fig. 3:**
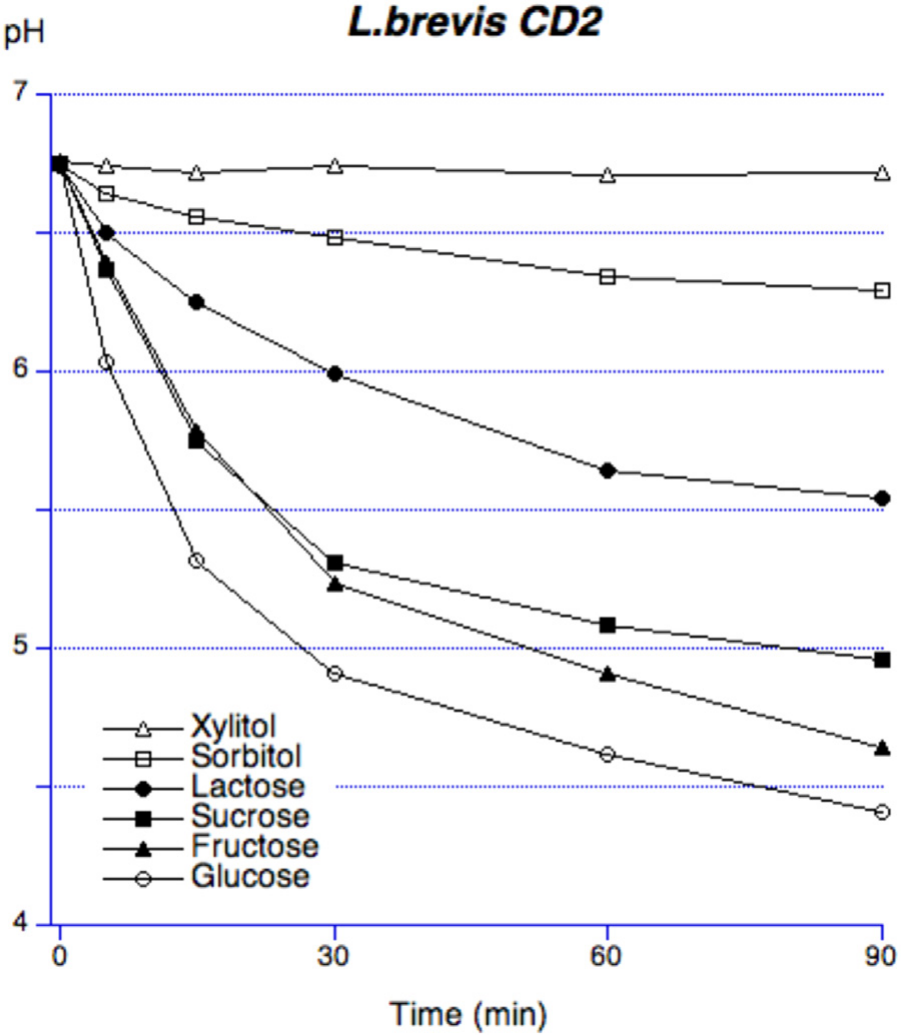
pH curves of different sugars added to suspensions of *L. brevis* CD2.

The evaluation of acid production with different sugar solutions when added to the suspension of *L. brevis* (Figure 3) showed that glucose resulted in the most pronounced pH fall, followed by fructose, sucrose, and lactose. Sugar alcohols caused only minor pH changes over time.

The baseline pH of dental plaque alone (about 5.75), was lower than of any other combinations of dental plaque with different bacterial strains (Figure 3). Regarding glucose, none of the bacterial strain inhibited acid production, although the pH decrease in *L. brevis* and *L. reuteri* was like that observed with dental plaque alone. The suspension containing *L. brevis* produced less acid after the addition of fructose, lactose, and sucrose, compared to the suspension of dental plaque. Moreover, the pH reduction for *L. plantarum* and *L. rhamnosus* after adding lactose, and for *L. reuteri* after adding fructose, was less pronounced than that of dental plaque suspension alone. The addition of *S. mutans* to the dental plaque suspension resulted in the highest pH falls. Sugar alcohols caused minimal pH changes during the 90-minute period.

### Ex vivo

When evaluating AUC and maximal pH drop, a similar pattern was observed (Table 2). The differences were statistically significant for most sugar groups (*P* < .05). Only xylitol showed no significant difference (*P* = .12) among the bacteria. The minimum pH drop showed statistically significant variations across all sugar groups (*P* = .05). *L. brevis* produced less acid after the addition of fructose, lactose, and sucrose compared to dental plaque. The ranking order for both AUC and maximal pH drop revealed that *L. brevis* had the least pronounced pH decrease.

**Table 2:** Area under the curve (AUC_5.7_) and maximum pH fall expressed as mean ± SD of the pooled plaque after sugar addition with the rank order of different bacterial solutions for respective sugar solution after 90 minutes. Two-way factorial ANOVA was performed.

AUC_5.7_ (mean± SD) Two-way factorial ANOVA	*number of observation 42 R-squared = 0.64 F = 4.25 P < .01*						
Bacterial strain		Glucose	Fructose	Sucrose	Lactose	Sorbitol	Xylitol
*Plaque* (Pl)		98.22 ± 1.24 (1)*	83.17 ± 27.71 (2)	103.20 ± 14.52 (2)	66.78 ± 12.76 (2)	10.77 ± 4.86 (5)	2.10 ± 3.12 (6)
Pl *+ L. brevis* CD2		125.20 ± 24.40 (2)	82.69 ± 31.40 (1)	100.70 ± 10.83 (1)	65.70 ± 22.41 (1)	1.04 ± 10.18 (1)	0.00 ± 0.3 (1)
Pl *+ L. reuteri* SDM 17938		133.10 ± 5.97 (3)	90.45 ± 17.22 (3)	119.80 ± 11.53 (3)	106.30 ± 6.36 (5)	4.95 ± 13.05 (3)	0.00 ± 1.68 (1)
Pl *+ L. plantarum* 931		158.80 ± 5.94 (5)	135.83 ± 19.82 (5)	149.14 ± 6.86 (5)	74.50 ± 20.17 (3)	8.57 ± 17.13 (4)	0.42 ± 2.09 (3)
Pl *+ L. rhamnosus* LB21		155.70 ± 13.54 (4)	124.1 ± 44.52 (4)	143.2 ± 8.71 (4)	75.95 ± 18.4 (4)	2.87 ± 13.15 (2)	1.29 ± 2.98 (5)
Pl *+ S. mutans* Ingbritt		166.30 ± 5.1 (6)	158.42 ± 19.45 (6)	165.42 ± 8.27 (6)	107.80 ± 11.97 (6)	14.09 ± 29.03 (6)	1.16 ± 4.83 (4)
*P*-value		<.05					

Maximum pH fall (mean ± SD) Two-way factorial ANOVA	*number of observation 42 R-squared = 0.67 F = 4.82 P < .01*						
Bacterial strain	Glucose		Fructose	Sucrose	Lactose	Sorbitol	Xylitol

*Plaque* (Pl)	1.42 ± 0.03 (1)†		1.29 ± 0.30 (1)	1.49 ± 0.16 (1)	1.09 ± 0.16 (1)	0.17 ± 0.07 (5)	0.14 ± 0.15 (6)
Pl *+ L. brevis* CD2	1.82 ± 0.08 (2)		1.32 ± 0.39 (2)	1.52 ± 0.08 (2)	1.09 ± 0.28 (1)	0.02 ± 0.33 (1)	-0.01 ± 0.05 (1)
Pl *+ L. reuteri* SDM 17938	1.88 ± 0.08 (3)		1.49 ± 0.16 (3)	1.80 ± 0.10 (3)	1.59 ± 0.07 (5)	0.08 ± 0.35 (2)	-0.01 ± 0.07 (1)
Pl *+ L. plantarum* 931	2.28 ± 0.08 (6)		2.05 ± 0.24 (5)	2.13 ± 0.02 (5)	1.19 ± 0.26 (3)	0.14 ± 0.40 (4)	0.06 ± 0.13 (3)
Pl *+ L. rhamnosus* LB21	2.26 ± 0.11 (4)		2.01 ± 0.50 (4)	2.07 ± 0.06 (4)	1.29 ± 0.24 (4)	0.08 ± 0.37 (2)	0.10 ± 0.21 (5)
Pl *+ S. mutans* Ingbritt	2.26 ± 0.04 (4)		2.18 ± 0.26 (6)	2.22 ± 0.10 (6)	1.66 ± 0.14 (6)	0.23 ± 0.64 (6)	0.06 ± 0.21 (3)
*P*-value	<.05						

⁎ Ranking order of AUC_5.7_ (mean ± SD) for respective sugar solution.

† Ranking order of maximal pH fall (mean ± SD) for respective sugar solution.

### In vivo

**Fig. 4:**
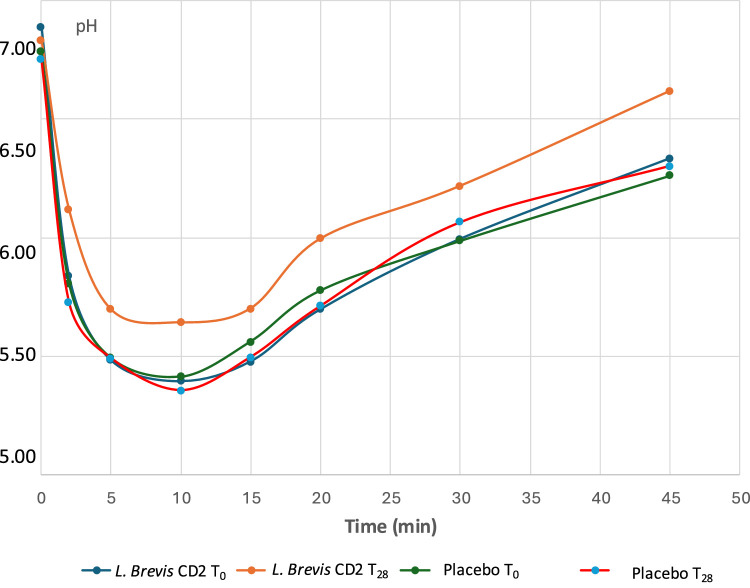
pH curves in the 2 groups (*L.brevis* CD2 and placebo) recording for 45 minutes at baseline (t_0_) and at follow-up (t_28_).

All volunteers completed the trial, and no adverse effects were reported from either lozenge. At the end of the probiotic period, a significant reduction in plaque acidogenicity was noted at time points 2, 5, and 45 minutes (*P* < .05) following the sugar rinse (Figure 4). In the comparison between the test and control lozenges, the lozenge containing *L. brevis* CD2 produced a statistically significant higher pH at 2 and 45 minutes (*P* < .05). AUC_5.7_ and maximal pH fall tended to decrease (*P* < .01), while AUC_6.2_ and minimal pH were unchanged following exposure to *L. brevis* CD2 (Table 3). The plaque microbiota showed considerable variation among the volunteers. The plaque index and the oral streptococci levels were significantly different between the 2 groups at the end of the trial (*P* < .01) (Table 4). Although the stimulated salivary secretion rate tended to increase at the end of the placebo period (*P* = .06), these changes were not significant (*data not shown*).

**Table 3:** Minimum pH value, maximum pH fall, area under the curve (AUC_5,7_ and AUC_6.2_) expressed as mean ± SD in the 2 groups *(Lb brevis* CD2 and placebo in dental plaque. Two-way factorial ANOVA analysis was performed.

		*Baseline*		*Follow-up (28dd)*		*Repeated Anova*
		*Lb brevis CD2*	Placebo	*Lb brevis CD2*	Placebo	*N = 56*
		*mean* ± SD	*mean* ± SD	*mean* ± SD	*mean* ± SD	
Min pH value		5.32 ± 0.42	5.39 ± 0.48	5.49 ± 0.53	5.2 ± 0.53	*F = 5.34 p < .01*
Max pH Fall		1.55 ± 0.48	1.41 ± 0.36	1.32 ± 0.49	1.52 ± 0.50	*F = 4.44 P < .01*
AUC_5.7_		6.65 ± 6.64	4.34 ± 4.48	6.46 ± 7.49	7.94 ± 9.59	*F = 1.04 P = .38*
AUC_6.2_		19.49 ± 13.18	12.56 ± 10.75	19.63 ± 14.12	20.17 ± 15.25	*F = 3.75 P < .01*

**Table 4:** Plaque index, percentage of *Streptococcus* mutans, *Lactobacillus*, and oral *Streptococci* in plaque, respect to bacteria total counts expressed as mean ± SD in the 2 groups *(Lb brevis* CD2 and placebo). Two-way factorial ANOVA analysis was performed.

	*Baseline*		*Follow-up (28dd)*		*Repeated Anova*
	*Lb brevis* CD2	Placebo	*Lb brevis* CD2	Placebo	*n = 56*
Plaque Index *(mean±SD)*	1.60 ± 0.50	1.29 ± 0.60	1.46 ± 0.42	1.58 ± 0.49	*F = 4.47****P < .01***
Bacteria *(%mean±_95_CI)*					
*S. mutans*	0.02 ± 0.0005	0.11 ± 0.006	0.12 ± 0.006	0.86 ± 0.05	*F = 1.62 P = .11*
*Lactobacilli*	0.05 ± 0.002	0.01 ± 0.0004	0.10 ± 0.004	0.01 ± 0.0003	*F = 1.22 P = .30*
Oral Streptecocci	35.00 ± 0.44	23.05 ± 10.84	19.99 ± 0.19	23.22 ± 0.34	*F = 2.06 P = .03*

The level of salivary mutans *Streptococci* showed a trend toward increased levels at the end of the test period (*P* = .09) and a statistically significant increase at the end of the placebo period (*P* < .05). Lactobacilli levels in saliva tended to increase at the end of the test period compared to the placebo period (*P* = .09). The total number of salivary streptococci and viable bacteria remained unchanged during either period.

When calculating the percentage of mutans *Streptococci* of the total streptococci and total viable count, the MS/TS ratio tended to increase at the end of both the test (*P* = .07) and placebo (*P* = .1) periods. The MS/TC ratio increased significantly at the end of both the test and placebo periods (*P* < .05). The lactobacilli/total viable ratio bacteria showed a significant increase at the end of the test period compared to the placebo period (*P* < .05).

No significant effect was found on the total viable count, MS/TC, LB/TC, and MS/TS ratios during either the test or placebo period. Nevertheless, total *Streptococci*/total viable bacteria ratio tended to decrease at day 28 of the test period (*P* = .07).

## Discussion

The present study evaluated the potential of *L. brevis* CD2 to either promote or inhibit acid production following sugar exposure. The null hypothesis stated that *L. brevis* CD2 would not modify acid production or plaque acidogenicity. The study's outcomes rejected this null hypothesis, supporting the significant potential of *Lactobacillus brevis CD2* in reducing acid production and plaque acidogenicity, both in vitro and in vivo*.*

Acidogenic lactobacilli are strongly associated with the caries process. Therefore, it is essential to investigate the suitability of administering *L. brevis* and other lactobacilli probiotic strains in caries prevention programs under controlled in vitro conditions with minimal risk for confounding factors.

Acid production inhibits the growth of pathogenic bacteria in the gastrointestinal tract.^31^ but it may potentially contribute to an increased risk of caries,^4^^,^^18^, ^19^, ^20^, ^21^^,^^32^^,^^33^ suggesting that acidogenic probiotic strains effective in the gastrointestinal tract may not be optimal for caries prevention.

The accuracy of the method used in this study might be considered high. The method's validity was supported by the minimal pH changes observed in the control solution (without sugar) during the 90-minute period when no sugar was added. The reliability of the method might be also considered high, as similar pH decreases were observed in all 3 test repetitions when glucose was added. The suspension of dental plaque and *S. mutans Ingbritt*, which served as a positive control, resulted in the greatest pH drop. The method's reliability was further confirmed by the minor pH changes observed in the control solution (without sugar) and stable readings from the pH electrode. Compared to lactic acid measurement, pH evaluation is a simpler, cost-effective method for monitoring bacterial fermentation activity over time.

Although all probiotic strains decreased pH following sugar exposure, the *L. brevis* and *L. reuteri* suspensions exhibited significantly less pronounced pH falls. Although all probiotics strains decreased pH following a sugar exposure, suspension of *L. brevis* and *L. reuteri* have significantly less pronounced pH fall. None of the bacterial strains tested fermented xylitol, which is consistent with previous findings.^1^^,^^2^^,^^4^^,^^34^ These results suggest that *L. brevis CD2* could be combined with other caries prevention strategies, such as the use of sugar alcohols.

The ability to inhibit acid production was dependent on both the probiotic strain and the type of sugar, as confirmed by a previous study.^32^^,^^33^ In the present study, the suspension of *L. reuteri* inhibited acid production compared to *L. reuteri*, following fructose exposure, consistent with previous research.^11^ However, *L. brevis* demonstrated even higher inhibitory capacity than *L. reuteri* when exposed to fructose. This is a significant finding considering the widespread consumption of fructose in modern diets.^35^ In addition, in this study, plaque samples were collected from subjects with a higher mean DMFT score compared to those enrolled in a previous paper,^11^ suggesting that a population with a higher caries risk could benefit more from probiotics in terms of caries prevention. The clinical implications of these findings are considerable. *L. brevis* CD2 tablets could be a valuable adjuvant in caries prevention, particularly for high-risk subjects, as previous studies in paediatric and diabetic populations have shown^20^^,^^21^ suggesting future potential applications in other high-risk groups such as elderly or subjects with various forms of disability.

However, there are a number of limitations to our study. The in vitro findings may not fully replicate the complexity of biofilm behaviour in vivo, and the relatively small sample size of the clinical trial requires larger studies to confirm the observed effects. Nevertheless, previous studies have shown that the administration of *L. brevis* led to a significant reduction in both plaque acidogenicity and salivary *mutans Streptococci* in high caries-risk schoolchildren.^20^ These results still require confirmation in an in vivo study. In addition, the long-term effects of *L. brevis* CD2 on oral health remain to be investigated, which is crucial for establishing its usefulness in preventive dentistry.

The high arginine deiminase activity of *L. brevis*, which converts arginine to citrulline and ammonia, provides energy for bacterial growth.^23^ Previous research has shown that arginine deiminase activity may contribute to the neutralisation of dental plaque acid following sugar catabolism by bacteria.^24^ This may explain the apparent higher acid inhibition capacity of *L. brevis* compared to other probiotic strains.

Future research should focus on evaluating the combined effects of *L. brevis CD2* with other probiotic strains or sugar alcohols, as well as its long-term efficacy in reducing caries risk in diverse populations. Investigations into its impact on other oral health indicators, such as halitosis and periodontal disease, would further elucidate its potential applications.

## Conclusions

In this study, it was observed that both acid production and inhibition are influenced by the specific probiotic strain and the type of sugar involved. In terms of acid production, *Lactobacillus reuteri* and *Lactobacillus brevis CD2* exhibited least pronounced pH decrease compared to the other bacteria tested. No strains fermented xylitol. In term of acid inhibition, *L. brevis CD2* demonstrated a notable effect when fructose, lactose, or sucrose were added, showing a reduced acidogenic response compared to dental plaque alone.

Furthernore,*Lactobacillus brevis* CD2 exhibited inhibitory effects on plaque acidogenicity in vitro, ex vivo, and in vivo, highlighting its potential use in caries prevention strategies.

## Conflict of interest

None disclosed.
